# Kinetic and structural studies on the interactions of *Torpedo californica* acetylcholinesterase with two donepezil-like rigid analogues

**DOI:** 10.1080/14756366.2018.1458030

**Published:** 2018-04-13

**Authors:** Rosanna Caliandro, Alessandro Pesaresi, Luca Cariati, Antonio Procopio, Manuela Oliverio, Doriano Lamba

**Affiliations:** a Istituto di Cristallografia, Consiglio Nazionale delle Ricerche, Trieste, Italy;; b Dipartimento di Scienze della Salute, Università degli Studi “Magna Graecia”, Catanzaro, Italy

**Keywords:** donepezil analogues, acetylcholinesterase, β-secretase-1, X-ray Crystallography, inhibition kinetics

## Abstract

Acetylcholinesterase inhibitors were introduced for the symptomatic treatment of Alzheimer’s disease (AD). Among the currently approved inhibitors, donepezil (DNP) is one of the most preferred choices in AD therapy. The X-ray crystal structures of *Torpedo californica* AChE in complex with two novel rigid DNP-like analogs, compounds **1** and **2**, have been determined. Kinetic studies indicated that compounds **1** and **2** show a mixed-type inhibition against *Tc*AChE, with *K_i_* values of 11.12 ± 2.88 and 29.86 ± 1.12 nM, respectively. The DNP rigidification results in a likely entropy-enthalpy compensation with solvation effects contributing primarily to AChE binding affinity. Molecular docking evidenced the molecular basis for the binding of compounds **1** and **2** to the active site of β-secretase-1. Overall, these simplified DNP derivatives may represent new structural templates for the design of lead compounds for a more effective therapeutic strategy against AD by foreseeing a dual AChE and BACE-1 inhibitory activity.

## Introduction

Alzheimer’s disease (AD) is a multi-factorial progressive neurodegenerative disorder, clinically characterized by age-related loss of memory and cognitive impairment.^1^ Cholinergic enzyme deficiency, increased accumulation of β amyloid (Aβ) in the senile plaque neurites, the formation of neurofibrillary tangles composed of a highly phosphorylated form of the microtubule-associated protein tau, oxidative stress, dyshomeostasis of biometals, mitochondrial abnormalities, and neuroinflammatory processes are among the major factors implicated in the multi-faceted pathogenesis of AD.^2^^,^^3^

Despite the extensive research in the field, AD pathogenesis is still at some extend obscure. Mechanisms linking AD with certain comorbidities, namely diabetes mellitus, obesity and dyslipidemia, are increasingly gaining importance, mainly due to their potential role in promoting AD development and exacerbation.^4^ Their exact cognitive impairment trajectories, however, remain to be fully elucidated. The most significant of these are: (i) the *cholinergic hypothesis* which postulates that the cognitive decline can be linked to a decrease in the amount of the neurotransmitter acetylcholine (ACh)^5^ and (ii) the *amyloid hypothesis* which instead ascribes AD symptoms to the Amyloid Precursor Protein (APP) that undergoes a sequential post-translational proteolysis/processing by β-secretase 1 (BACE-1) and γ-secretase leading to the formation of hydrophobic Aβ peptide fibrils that readily accumulate and deposit on neuritic plaques in the gray matter of the brain.^6^^,^^7^

Acetylcholinesterase (AChE) has been shown to bind to Aβ and to play a role in the formation of Aβ plaques.^8^ ACh is also broken down by butyrylcholinesterase (BChE) to a lesser extent and at slower rate, although its activity progressively increases in patients with AD, while AChE activity remains unchanged or declines.^9^

To date, no available treatment is known to stop the progression of AD. The cholinesterase inhibitors donepezil (DNP), galantamine, and rivastigmine and the *N*-Methyl-D-Aspartate (NMDA) receptor antagonist memantine which works by regulating the activity of glutamate, an important neurotransmitter in the brain involved in learning and memory, are currently prescribed for the treatment of mild-moderate AD.^10^^,^^11^ Although AChE inhibitors are not able to halt the progress of the disease, they only seem to act as palliative by temporary ameliorating cognitive impairment, these drugs improve nonetheless the quality of life for patients and caregivers.^12^^,^^13^

**Figure 1.**
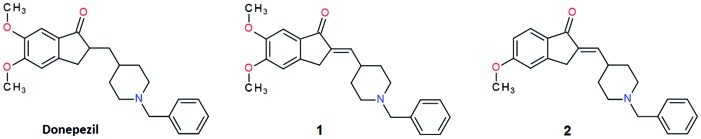
Structural formulas of donepezil and donepezil-analogs **1** and **2**.

Among AChE inhibitors, DNP (Figure 1) is the most preferred because it gives the most positive response in AD treatment and has some advantages as blood-brain barrier permeability, non-hepatotoxicity, the least side effect and usage once-daily.^14^

On a quest to develop new and effective bioactive chemical entities, **DNP** structurally related inhibitors,^15–22^ including the synthesis and biological evaluation of hybrid inhibitors^23–29^ aiming to expand the multi-target profile of this lead compound, have been the subject of extensive structure–activity relationship studies seeking at the simultaneous (i) inhibition of AChE catalytic function; (ii) anti-aggregating activity on both AChE-induced and self-mediated Aβ-aggregation; and (iii) inhibition of BACE-1, the steady hunt for an effective disease-modifying treatment.^30–32^

It has been reported that compounds with a double C–C bond between the indanone core of **DNP** and the phenyl-*N*-methylbenzylamino moiety of 3-{4-[(benzylmethylamino)methyl]phenyl}-6,7-dimethoxy-2H-2-chromenone (AP2238),^33–35^ the first compound published to bind both anionic sites of AChE allowing the simultaneous inhibition of the catalytic and the Aβ pro-aggregating activities of AChE, retain the **DNP** potency against AChE and display a promising BACE-1 inhibition profile thanks to their increased structural rigidity.^32^

On a large scale, **DNP** originally had been synthesized from alkylidene or arylidene-2-indanone formed by aldol condensation chemistry as key intermediates followed by catalytic reduction.^36^ The process suffered from several disadvantages, i.e. the use of unacceptable solvent such as hexamethyl phosphoric amide, the formation of side products during catalytic reduction and the need of column chromatography to remove the unwanted side products. Therefore, several viable and efficient synthetic routes had been developed that offer cost reduction as well as avoiding the use of hazardous reagents.^37–39^

A synthetic pathway for **DNP** analogs through eco-friendly synthetic procedures has been recently reported in order to improve yields, regio-selectivity and rate of each synthetic step and to reduce the coproduction of waste at the same time.^40^ The synthesized derivatives were designed in order to study the influence of the characteristic unsaturation between the 1-indanone and the N-benzylpiperidine-4-carboxaldehyde synthons on **DNP***in vitro* inhibitory activity of human erythrocytes and *Electrophorus electricus* AChE, horse serum BChE and mouse BACE-1.

Two potential new lead compounds, **1** and **2** (Figure 1), as promising simplified **DNP** analogs, were envisaged which display better dual activity and IC_50_ values against both AChE and BACE-1 enzymes, if compared to structurally related molecules.^18^^,^^32^

We undertook a detailed kinetic study of the *Torpedo californica* AChE (*Tc*AChE) inhibition mechanism by compounds **1** and **2** supported by a thorough crystallographic analysis, comparing the presently reported X-ray crystal structures of *Tc*AChE in complex with **1** and **2**, respectively, with the X-ray crystal structure of *Tc*AChE–**DNP** complex previously determined.^41^ The characterization of the complexes unveiled the structural basis for the modulation of AChE inhibitory activity as a consequence of the introduced rigidity in **DNP**.

This information provides the basis for a structure guided approach to the development of simplified **DNP** inhibitors more potent and more selective towards either AChE and BACE-1.

## Materials and methods

### Kinetic analysis of TcAChE inhibition

The enzymatic activity of *Tc*AChE was evaluated spectrophotometrically at room temperature by Ellman’s method^42^ using a GE Ultrospec 7000 double beam spectrophotometer. The rate of increase in the absorbance at 412 nm was followed for 5 min. The assay solution consisted of K-phosphate buffer at pH 7.0, 340 µM 5,5′-dithiobis(2-nitrobenzoic acid) (Sigma-Aldrich, Milan, Italy) and 4.5 ng/mL of enzyme. The reaction was initiated by addition to the reaction mixture of the substrate acetylthiocoline (Sigma-Aldrich, Milan, Italy). To gain insights into the mechanism of action of **1**, **2** and **DNP**, reciprocal plots of 1/velocity *versus* 1/[substrate] were constructed at substrate concentration in the 10–200 µM range. Data points are average values of three replicates. Three concentrations of inhibitors were selected for this study: 5, 10, and 20 nM. The plots were assessed by a weighted least-squares analysis that assumed the variance of the velocity (*v*) to be a constant percentage of *v* for the entire data set. Calculation of the inhibitor constant (*K_i_*) value was carried out by re-plotting slopes of lines from the Lineweaver–Burk plot versus the inhibitor concentration and *K_i_* was determined as the intersect on the negative *x*-axis. The apparent *K_i_*’ (dissociation constant for the enzyme–substrate–inhibitor complex) value was determined by plotting the apparent 1/*v*_max_ versus inhibitor concentration. Data analyzes were performed with Graph 4.4.2.

### Crystallization, X-ray data collection, and refinement

*Tc*AChE was isolated, purified and crystallized as previously described,^43^ except for the affinity chromatography ligand, mono-(aminocaproyl)-p-aminophenyltrimethylammonium. Owing to its relatively limited solubility in water, **1** or **2** were dissolved in DMSO (100 mM). The crystals of the complexes were obtained by soaking native crystals at 4 °C for 24 h, in 2 mM **1** or **2**, 30% PEG [poly(ethylene glycol)] 200, 8% DMSO, 100 mM MES [2-(N-morpholino)ethanesulfonic acid] at pH 6.2.

X-ray diffraction data were collected at the XRD-1 beamline of the Italian Synchrotron Facility ELETTRA (Trieste, Italy).^44^ A PILATUS 2 M detector (Dectris Ltd., Baden, Switzerland) and focusing optics were employed for the measurements. The crystals were flash-cooled in a nitrogen stream at 100 K, using an Oxford Cryosystems cooling device (Oxford, UK). Data processing was done with *MOSFLM* version 7.0.7 (Cambridge, UK)^45^^,^^46^ and the CCP4 package version 6.3.0 (Didcot, UK).^47^

The enzyme−ligands crystal structures were determined by Patterson search methods with the *PHASER* package version 2.3.0^48^ using as search model the refined coordinates of the *Tc*AChE – methylene blue with PEG complex (PDB ID 5E4T)^49^ after removal of the ligands and the water molecules, respectively.

Crystallographic refinement of the complexes were performed with *REFMAC* version 5.7.0032.^50^ All data within the resolution range were included with no-σ cutoff. Bulk solvent correction and anisotropic scaling were applied. The Fourier (2|*F*_o_| − |*F*_c_|, ϕ_c_) and (|*F*_o_| − |*F*_c_|, ϕ_c_) maps were computed with σ-A weighted coefficients^51^ after initial refinement of the native protein structure (without ligand and water molecules) by rigid body followed by maximum likelihood positional and individual isotropic temperature factor refinements. Prominent electron density features along the catalytic gorges of the *Tc*AChE-**1** and *Tc*AChE-**2** complexes, respectively allowed the unambiguous fitting of the ligands **1** and **2**. Carbohydrates (*N*-acetyl β-d-glucosamine, α-d-mannose, α-L-fucose and β-d-mannose linked at Asn59, Asn416 and Asn457) were built in by inspecting electron density maps. Peaks in the difference Fourier maps that were greater than 1.8 r.m.s.d. were automatically added as water molecules to the atomic model and retained if they met stereochemical requirements, and their B factors were less than 70 Å^2^ and 75 Å^2^ in *Tc*AChE-**1** and *Tc*AChE-**2,** respectively, after refinement. Maps inspection and model corrections during refinement were based on the graphics program *COOT* version 0.7.^52^

Atomic coordinates and structure factor amplitudes of the *Tc*AChE-**1** and *Tc*AChE-**2** complexes have been deposited in the Brookhaven Protein Data Bank under the PDB ID codes 5NAP and 5NAU, respectively.

### Computational docking simulations

Molecular docking studies were performed using the AutoDock 4.2 package.^53^ The X-ray structure of human BACE-1 (*h*BACE-1) in complex with SCH734723 (PDB ID 2QP8)^54^ was used as template. PDB files of the ligands were generated using the PRODRG server^55^ and the The AutoDock Tool (ADT) was used to assign atomic partial charges and to convert the target protein and ligands structures to the required PDBQT format.

The grid box (with dimensions *X* = 40, *Y* = 60, *Z* = 40 points and spacing between the grid points of 0.375 Å) was centered on the coordinate *X* = 16.1, *Y* = 1.6, and *Z* = 15.7, in order to cover the entire favorable BACE-1 binding site. Potential maps were generate with the AutoGrid feature. For each ligand 50 runs of Monte Carlo simulated annealing were carried out (for each run 250 annealing cycles were performed; 25,000 moves were accepted and 25,000 moves were rejected).

The AutoDock semi-empirical force field includes intramolecular terms, a “full” desolvation model, and also considers directionality in hydrogen bonds. The conformational entropy is calculated from the sum of the torsional degrees of freedom. Water molecules are not modeled explicitly though, but pair-wise atomic terms are used to estimate the water contribution (dispersion/repulsion, hydrogen bonding, electrostatics, and desolvation), where weights are added for calibration (based on experimental data). The theoretical protein-ligand binding energy ΔG_b_ includes the calculation of i) the energy of ligand and protein in the unbound state; ii) the energy of the protein-ligand complex. Then the difference is computed: ΔG_b_= (V_bound_^L-L^ – V_unbound_^L-L^) + (V_bound_^P-P^ − V_unbound_^P-P^) + (V_bound_^P-L^ − V_unbound_^P-L+^Δ*S_conf_*) where *P* refers to the protein, *L* refers to the ligand, *V* are the pair-wise evaluations (see above) and Δ*S_conf_*denotes the loss of conformational entropy upon binding.^56^

## Results

### X-ray crystal structure of *Tc*AChE-1 and *Tc*AChE-2 complexes

**Figure 2.**
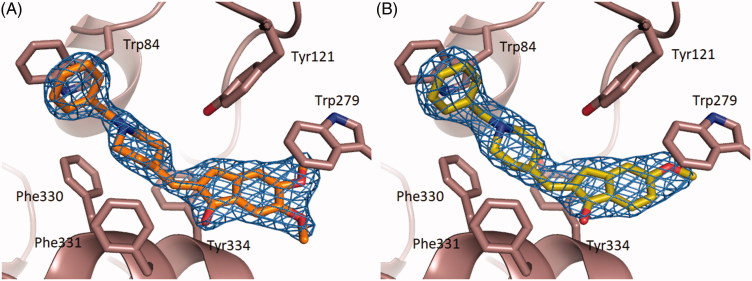
Close-up view of the active site of the *Tc*AChE-**1** (**A**) and (**B**) *Tc*AChE-**2** complexes. The final (2|*F*_o_| − |*F*_c_|, ϕ_c_) σA-weighted electron density map is contoured at 1.5σ. **1** and **2** are shown as stick models with carbon, oxygen and nitrogen atoms colored orange, red and blue, respectively. Selected key protein residues (Cα atoms and side chains) in the vicinity of **1** or **2** are rendered in stick format and labeled appropriately. Hydrogen bonding interactions and water molecules have been omitted for clarity. Created using PyMOL.^57^

The X-ray crystal structures of *Tc*AChE-**1** and TcAChE-**2** complexes have been determined and refined at 2.17 Å and 2.25 Å resolution, respectively (Figure 2(A,B)).

The crystal parameters, data collection, and refinement statistics are summarized in Table 1.

**Table 1.** Summary of crystallographic data of the *Tc*AChE – 1 and *Tc*AChE – 2 complexes.

Data collection	*Tc*AChE – **1**	*Tc*AChE – **2**
X-ray source	XRD-1, 5.2 R ELETTRA, Trieste (Italy)	
Wavelength (Å)	1.00	
Detector	Pilatus 2 M – Dectris Ltd.	
Space group	P3_1_21	
Unit cell parameters		
a,b (Å)	111.53	111.62
c (Å)	136.88	136.71
Mosaicity (°)	0.80	0.56
Resolution range (Å)	78.92 − 2.17 (2.29 − 2.17)^a^	48.33 − 2.25(2.37 − 2.25)^a^
Number of measurements	306,806	329,107
Number of unique reflections (I ≥ 0)	52,442 (7560)	47,111 (6824)
Completeness (%)	100.0 (100.0)	99.8 (99.9)
Multiplicity	5.9 (5.8)	7.0 (6.4)
<I/σ (I)>	9.2 (3.1)	4.7 (1.5)
*R*_merge_^b^	0.119 (0.526)	0.196 (0.860)
*R*_pim_^b^	0.053 (0.254)	0.077 (0.363)
*R*_meas_^b^	0.130 (0.618)	0.211 (0.937)
CC_1/2_	0.994 (0.891)	0.986 (0.848)
CC*	0.998 (0.971)	0.993 (0.918)
Refinement statistics		
Resolution range (Å)	55.90 − 2.17	48.33–2.25
Number of reflections (*F*_o_ ≥ 0)	49,664	44,654
*R*_all_^c^	0.171 0.189	
*R*_work_^c^	0.170	0.187
*R*_free_^d^	0.210	0.228
Number of atoms		
Non-hydrogen protein	4212	4205
Non-hydrogen waters	425	279
Non-hydrogen ligand	28 26	
Non-hydrogen carbohydrates	124	124
Rmsd bond lengths/bond angles (Å, °)^e^	0.021/2.0	0.020/1.9
Ramachandran plot (%) favored/allowed regions (%)^f^	94.5/5.5	93.8/6.2
Average temperature factors (Å^2^)		
Protein	29.3	37.1
Water	39.7	40.9
Non-hydrogen ligand	39.5	58.8
Carbohydrates	75.9	84.5
Rmsd ΔB (Å^2^)^g^	3.51	3.95

^a^Number in parentheses refer to the highest resolution shell.

^b^*R*_merge_ = ∑**_h_**∑*_i_*‖I**_h_***_i_* − <I**_h_**>‖/∑**_h_**∑*_i_* I**_h_***_i_*_,_ with I**_h_** is the *i*th measurement of reflection **h**, and < I**_h_**> is the (weighted) average of all symmetry-related or replicate observations of the unique reflection **h**. The summations include all “*n*” observed reflections; *R*_pim_* =* ∑**_h_**(1/*n* – 1)^1/2^∑*_i_*‖I**_h_***_i_* − <I**_h_**>‖/∑**_h_**∑*_i_* I**_h_***_i;_ R*_meas_* =*∑**_h_**(n/n−1)^1/2^∑*_i_*‖I**_h_***_i_* − <I**_h_**>‖/∑**_h_**∑*_i_* I**_h_***_i_*.

^c^*R*_work_ = ∑**_h_**‖*F_o_*‖− ‖*F_c_*‖/∑**_h_**‖*F_o_*‖, where ‖*F_o_*‖ and ‖*F_c_*‖ are the observed and calculated structure factor amplitudes for reflection **h**. The summation is extended over all unique reflections to the specified resolution.

^d^*R*_free_, R factor calculated using 2705 (*Tc*AChE-**1**) / 2365 (*Tc*AChE-**2**), respectively, randomly chosen reflections (5%) set aside from all stages of refinement.

^e^Stereochemical criteria are those of Engh and Huber.^58^

^f^ The reliability of the protein structure has been assessed using the MolProbity package.^59^

^g^Rmsd ΔB (Å^2^) is the rms deviation of the B factor of bonded atoms (all atoms).^60^

**Figure 3.**
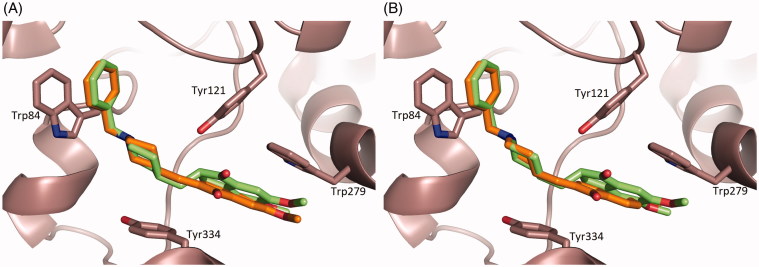
Superimposition of the crystal structure of the *Tc*AChE-Donepezil complex^41^ (carbon atoms colored in green) with the *Tc*AChE-**1** (**A**) and *Tc*AChE-**2** (**B**) complexes (carbon atoms colored in orange). Ligands and some of the active site key residues are shown as sticks with oxygen and nitrogen atoms colored red, and blue, respectively. Created using PyMOL.^57^

The binding conformation of **1** and **2** in the active-site gorge of *Tc*AChE closely mimic that being displayed by **DNP** (Figure 3(A,B)), extending from the bottom of the anionic subsite, near Trp84, to the peripheral anionic site, near Trp279 (PDB ID 1EVE).^41^

Interestingly, as **DNP**, neither **1** and **2** directly interact with the *Tc*AChE catalytic residue Ser200, nor with residues of the oxyanion hole.

As observed in the *Tc*AChE–**DNP** complex, one face of the benzyl ring of **1** and **2** displays a parallel π–π stacking against the six-membered ring of the indole moiety of Trp84 similarly to tacrine.^61^

The ring-to-ring distances average in *Tc*AChE-**1** and *Tc*AChE-**2** to 4.0 Å and in *Tc*AChE–**DNP** to 3.9 Å. On the opposite face, water molecule W1160 that in the crystal structure of the *Tc*AChE–**DNP** complex makes a classic aromatic hydrogen bond, appeared instead to be absent in the crystal structures of both the *Tc*AChE-**1** and *Tc*AChE-**2** complexes.

Donepezil, compounds **1** and **2** have a basic character containing a tertiary amine group. The pKa of **DNP** is 8.94 ± 0.18.^62^ Hence at pH 6.2 (see crystallization conditions) the piperidine ring of **1** and **2** are most likely protonated.

In the constricted region, halfway up the gorge, the charged nitrogen of the piperidine ring makes a cation–π interaction^63^ with the phenyl ring of Phe330.

**Figure 4.**
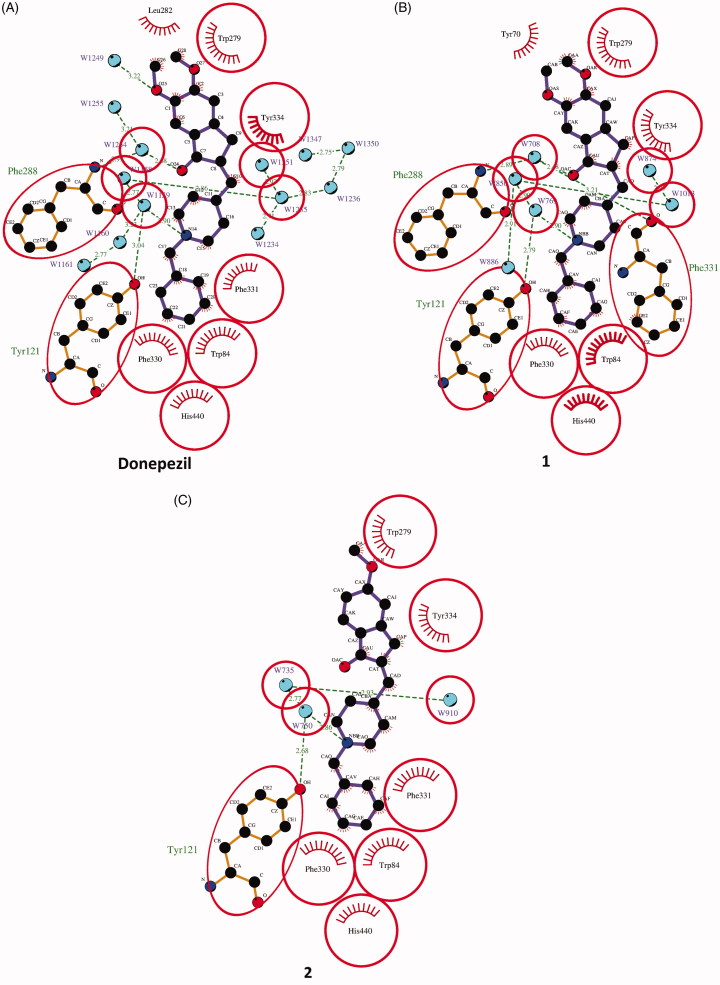
LigPlot + diagrams^64^ illustrating the *Tc*AChE–ligand interactions. Hydrophobic interactions are represented by red spokes radiating towards the ligand atoms they contact. Ligands are represented in purple. C, N, O, and atoms are represented in black, blue, and red, respectively. Water molecules are colored in cyan. The equivalent residues in *Tc*AChE-donepezil, *Tc*AChE-**1** and *Tc*AChE-**2** are shown on the plots by red circles.

The average distances are of 4.4 Å in *Tc*AChE-**1**, 4.5 Å in *Tc*AChE-**2** and 4.3 Å in *Tc*AChE-**DNP**. The ring nitrogen is engaged in an in-line H-bond with water W850 in *Tc*AChE-**1** and W750 in *Tc*AChE-**2** (Figure 4). This water molecule is present also in the crystal structure of the *Tc*AChE-**DNP** complex (W1159) at the identical H-bond distance of 2.9 Å, and in turn makes additional H bonds with Tyr121 OH, W1160 and W1158. Only the latter water is structurally conserved in *Tc*AChE-**1** (W769) and in *Tc*AChE-**2** (W735) whereas the former is absent in both complexes.

At the top of the gorge, the indanone ring stacks against the six-membered ring of the indole moiety of Trp279, in the peripheral binding site, in a classic parallel π–π interaction. The fact that the binding of **DNP**, **1** and **2** are strongly dependent on interaction with Trp279 and Phe330, which are absent in BChE, may explain their reported high relative specificity for AChE versus BChE.^40^

The carbonyl function on the indanone is not in direct contact with the protein, but in the *Tc*AChE–**DNP** and *Tc*AChE-**1** complexes, water molecules W1254 and W708, respectively, appear to make a structurally conserved water-bridged contact with the main chain NH of Phe288. The equivalent water in *Tc*AChE-**2** is instead absent (Figure 4).

Furthermore, in the *Tc*AChE-**DNP**, water molecule W1249 which is absent in both the *Tc*AChE-**1** and *Tc*AChE-**2** complexes, lies in the plane of the indanone moiety, and is H-bonded to one of the two methoxyl groups of **DNP** (Figure 4).

Significant differences can be noticed at the level of the π–π stacking interactions between the indanone ring and the peripheral anionic site residue Trp279, as a direct consequence of the introduced structural rigidity in **DNP**. The indanone ring position/orientation in *Tc*AChE-**1** and *Tc*AChE-**2** complexes result in an increased interfacial distance that mainly affects the indole moiety of Trp279 (Figure 3(A,B)) and in part the aromatic rings of Phe331 and Phe290. The average distances between the closest indanone and Trp indole aromatic carbons are 4.1 Å and 4.2 Å in *Tc*AChE-**1** and in *Tc*AChE-**2**, respectively, while the same distance averages to 3.7 Å in *Tc*AChE-**DNP**. In the latter complex, the indanone carboxyl interacts *via* edge-on van der Waals contacts with the aromatic rings of Phe290 and Phe331 at a distance of 3.5 Å and 3.1 Å, respectively. In *Tc*AChE-**1** and in *Tc*AChE-**2** these distances are 4.8 Å and 3.5 Å, and 5.0 Å and 3.7 Å, respectively. On the other hand **1** and **2** appear to be slightly closer to Tyr334 (Figure 3(A,B)), with an average distance between the indanone ring of **1** and **2** and the Tyr334 aromatic ring of 4.7 Å, whereas in *Tc*AChE-**DNP** this distance results to be 5.3 Å.

### Inhibition studies of DNP and compounds 1 and 2 on *Tc*AChE

**Figure 5.**
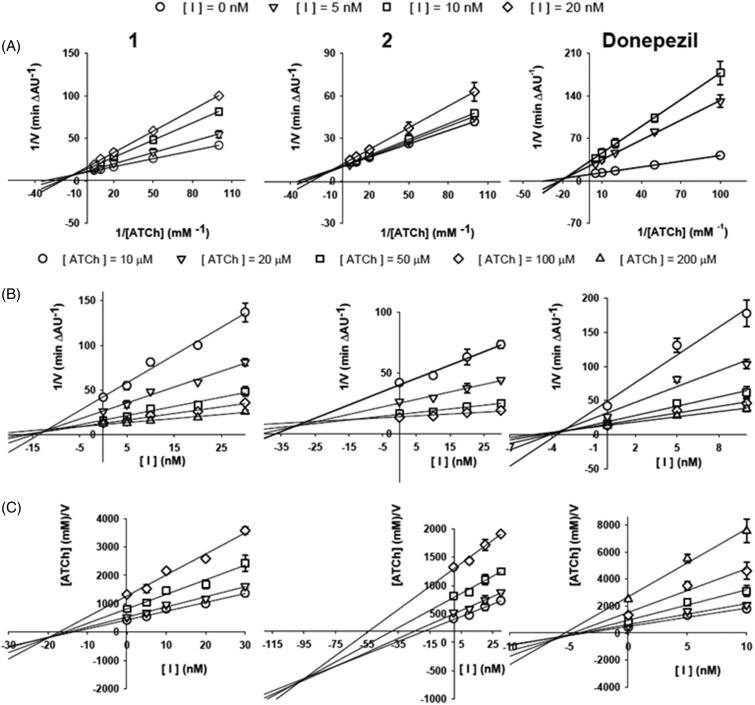
Kinetic study of *Tc*AChE inhibition by compounds **1**, **2** and Donepezil. (**A**) Overlaid Lineweaver-Burk reciprocal plots of the *Tc*AChE initial velocity (V) at increasing substrate (acetylthiocholine, ATCh) concentrations in the absence and in the presence of inhibitors (0−20 nM). (**B**) Dixon plots of 1/V against different concentration of inhibitors [I] at various concentrations of substrate ([ATCh] = 0–200 mM). (**C**) Cornish–Bowden plots of [ATCh]/V against inhibitor concentration[I] at various substrate concentrations. Data points are average values of three replicates. Lines were derived from a weighted least-squares analysis of the data points.

The mechanism of *Tc*AChE inhibition was investigated for the three inhibitors by building a linear Lineweaver-Burk double reciprocal plot (Figure 5(A)). The plots show that for all the investigated compounds, at increasing inhibitor concentrations corresponded an increase of both slopes (decreased *V*_max_) and intercepts (higher *K_m_*), a trend that is generally ascribed to a mixed-type inhibition. The mixed mechanism of action was also confirmed by the Dixon and the Cornish-Bowden plots (Figure 5(B,C)), that were used to determine, respectively, the inhibitor dissociation constants *K*_i_ and the dissociation constant for the enzyme–substrate–inhibitor complex *K*_i_′. The kinetic parameters measured for all the inhibitors are reported in Table 2. As already reported^40^ for the inhibition of the human erythrocytes AChE, **DNP** resulted to be a more potent *Tc*AChE inhibitor, *K_i_* = 2.98 ± 0.54 nM than its rigid **1** and **2** derivatives. Although **1** and **2** share a similar interaction with the *Tc*AChE active site, the presence of a second methoxy substituent on the indanone moiety of **1**, as in **DNP**, allows a better stacking against Trp279 accounting therefore for its slightly higher inhibitory potency compared to **2**, being the *K_i_* values of 11.12 ± 2.88 nM versus 29.86 ± 1.12 nM. In more detail, the methoxy substituent at position 5 of the indanone ring of the 5,6-dimethoxy compound **1** stacks on the benzene ring of Trp279 at a distance of 3.9 Å. A poorer stacking interaction, reflected by a distance of 4.2 Å, instead has been observed for the mono-methoxy compound **2**, substituted only at position 5 of the indanone ring.

**Table 2.** Inhibition constants for Donepezil and compounds **1** and **2** on the activity of *Tc*AChE. *K*_i_ is the dissociation constant for free enzyme; *K*_i_*’* is the dissociation constant for the enzyme-substrate-inhibitor complex.

	*K*_i_ (nM)	*K’*_i_ (nM)
Donepezil	2.98 ± 0.54	5.83 ± 0.76
**1**	11.12 ± 2.88	20 ± 1.04
**2**	29.86 ± 1.12	92.91 ± 10.12

### Molecular docking of *h*BACE-1 in complex with DNP and with compounds 1 and 2

**Figure 6.**
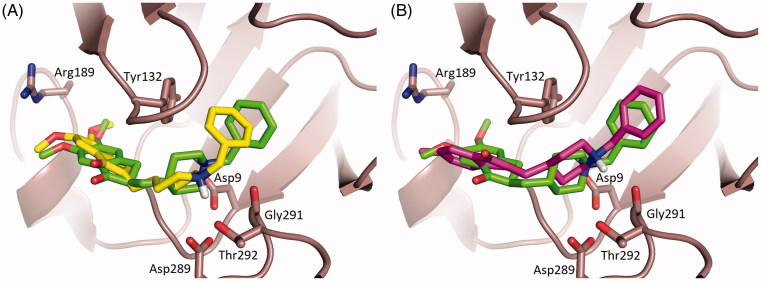
Superimposition of the top ranked docking poses of *h*BACE-1 in complex with Donepezil and **1** (**A**), and **2** (**B**), respectively. Donepezil, **1** and **2** are shown as stick models with carbon atoms colored green (Donepezil), yellow (**1**) and magenta (**2**) and oxygen and nitrogen atoms colored red and blue, respectively. Selected key protein residues (C_α_ atoms and side chains) in the vicinity of Donepezil or **1** or **2** are rendered in stick format and labeled appropriately. Created using PyMOL [57].

**DNP** and compounds **1** and **2** have been shown to inhibit *h*BACE-1 with similar potency (Table 3).^40^ Docking simulations pinpoint binding to the active site of BACE-1 to be mainly stabilized by the interactions between the methoxy substituents of the indanone and the benzyl moiety of the ligands with BACE-1 residues Arg189 and Tyr132, respectively (Figure 6).

**Table 3.** IC_50_ for mouse BACE-1 inhibition and theoretical binding energies.

	IC_50_ (nM)	ΔG_b_ (kcal/mol)
Donepezil	143	–10.70
**1**	697	–9.82
**2**	333	–9.84

Furthermore in **1** and **2** the protonated piperidine nitrogen is engaged in hydrogen networking to Thr292 and the catalytic residue Asp89 side chains, respectively, (**1**) or to the Gly291 main chain (**2**). In **DNP**, this molecular interaction is absent: the flexible junction between the indanone and the piperidinium moieties induce a *ca*. 120° twist of the N-benzylpiperidinum moiety with respect to the rigidified **DNP** analogs, **1** and **2**. As a consequence the protonated nitrogen of **DNP** seems to be no stabilized by specific interactions. The closer spatial vicinity between the benzyl ring of **DNP** and BACE-1 Tyr132 residue results nevertheless in a computed overall lower binding free energy (*ca*. 1.0 kcal/mol), in good agreement with the experimentally determined IC_50_ values (Table 3).

## Discussion

Compounds **1** and **2** are rigid **DNP** derivatives synthesized by using an innovative eco-friendly synthetic procedure.^40^ Both molecules have been selected as promising candidates for the development of drugs with dual activity on AChE and BACE-1.^40^ The C–C double bond that in both ligands links the indanone core to the *N*-benzyl piperidine moiety lower the flexibility of the molecules compared to **DNP**. Based on previous studies, it has been proposed that such structural rigidity can be an essential requirement to display inhibitory activity on BACE-1.^32^^,^^40^

So far the interaction between AChE and **DNP** derivatives characterized by the insertion of a double bond between indanone and N-benzyl piperidine, have only been broadly analyzed by docking studies and *in vitro* inhibitory assays.^32^^,^^40^ Here, for the first time we report the X-ray crystallographic structural analysis of *Tc*AChE in complex with two members of this class of compounds.

Although similar, the interaction with the *Tc*AChE active site of **DNP** and its rigid derivatives, compounds **1** and **2**, unveiled some differences that provide an explanation for the slightly better *Tc*AChE inhibitory potency exhibited by **DNP**. The N-benzylpiperidine moiety adopts an almost identical position/orientation within the enzyme catalytic gorge in the crystal structures of the three *Tc*AChE–ligand complexes. The rigidity introduced by the double C–C bond compels the indanone moiety of **1** and **2** to a somewhat less effective interaction with the peripheral anionic binding site residue Trp279 at the entrance of the catalytic gorge if compared to the *Tc*AChE–**DNP** complex. For **DNP** both the stacking of the aromatic ring of indanone against the indole ring of Trp279 and the edge-on van der Waals contacts between the indanone carbonyl with the aromatic rings of Phe331 and Phe290 take place at a significantly shorter distances than those observed in the *Tc*AChE-**1** and *Tc*AChE-**2** complexes.

The binding of **DNP** to *Tc*AChE is known to displace from the enzyme catalytic gorge mostly unbound solvent molecules, in fact only 5 (W625, W678, W679, W755, W767) out of the 25 conserved water molecules present in the active site of the native not inhibited *Tc*AChE crystal structure (PDB ID 2ACE)^65^ are lost upon **DNP** binding^66^, while two “novel” water molecules (W1249, W1351), absent in the native not inhibited *Tc*AChE crystal structure are stabilized due to bridging between the inhibitor and the enzyme.^41^ The binding of **1** displaces 8 of the conserved water molecules (W742, W749, W795, W628, W625, W678, W679, W767) in the native not inhibited *Tc*AChE crystal structure and one new molecule (W874) is stabilized. Overall, according to the crystallographic structures, the binding of **1** displaces from the *Tc*AChE gorge a larger number of solvent molecule than **DNP**, i.e. 7 versus 3. The net number of water molecules displaced from the native not inhibited crystal *Tc*AChE structure by the binding of **2** is 9 (W742, W749, W795, W628, W625, W678, W679, W767, W642).

These observations suggest that the introduction of the unsaturation in the **DNP** molecule might cause enthalpy–entropy ligand-binding compensation effects. The increased rigidity of **1** and **2** in fact, on the one hand, results in a weaker interaction with *Tc*AChE residues lining the catalytic gorge, highlighted by the lower number and weaker ligand–protein contacts, on the other should favor their binding due to a more favorable desolvation effect and a smaller loss of torsional entropy with respect to **DNP**. As a net result, despite ligands **1** and **2** do not optimally fill the volume of the enzyme catalytic gorge, if compared to **DNP**, the observed *Tc*AChE inhibition constants indeed confirm **1** and **2** to be marginally weaker inhibitors than **DNP**.

As the crystal structures of the *Tc*AChE complexes with compounds **1** and **2** reveal negligible differences in protein-inhibitor contacts, with respect to the *Tc*AChE–**DNP** complex we conclude that solvent effects contribute significantly to binding affinities.

The inhibition of human AChE by a series of **1** derivatives featuring different substituents on the phenyl moiety has been recently reported.^18^ The crystal structures of *Tc*AChE in complex with **1** and **2** provide a good explanation for the observed activities. Substituents in position 4 of the phenyl ring always lead to a poorer inhibition because they compromise the optimal stacking of the ligand on Trp279 at the peripheral anionic site. Given the little room available in the acyl pocket of AChE, only small substituent are acceptable in position 2 and 3 of the phenyl moiety. Fluorine in position 2 for instance leads to a 4-fold decrease of the IC_50_ while the bulkier methyl group or bromine cause a slightly weaker inhibition. An exception to this rule is the presence of a nitro group in position 3, which does not affect negatively the inhibition likely because of the stabilization of its negatively charged oxygen atoms by residues defining the enzyme oxy-anion hole, i.e. Gly118, Gly119, and Gly201.

Analogously to **DNP**, **1** and **2** showed a *Tc*AChE mixed-type enzyme inhibition. The currently determined **DNP** inhibition constants *K*_i_ and *K*_i_*’* of 2.98 ± 0.54 and 5.83 ± 0.76 nM, respectively, are in excellent agreement with those previously reported of 3.1 and 4.0 nM.^67^

Furthermore the observed *Tc*AChE *K*_i_ values well compared to those reported for the human erythrocyte AChE (*h*AChE) inhibition by **DNP**, **1** and **2**.^40^ These findings strongly suggest that the present protein–ligand interaction determinants based on *Tc*AChE can likely be extended to *h*AChE as well. The overall sequence identity/homology of *Tc*AChE versus *h*AChE are 57 and 74%, respectively. The identity/homology of *Tc*AChE versus *h*AChE significantly increase to 77 and 93%, respectively, if only the 43 residues defining and lining the catalytic gorge are considered.

Molecular docking simulations of **DNP** and of its rigidified analogs **1** and **2** demonstrated negative binding energies for *h*BACE-1, indicating good affinities towards the active site of the enzyme, in agreement with the *in vitro* IC_50_ values.^40^

Overall, the present kinetic, structural and computational docking studies pinpoint to the simplified **DNP**-like analogs, **1** and **2** as a new structural template for the design and optimization of lead compounds for a more effective therapeutic strategy against AD by foreseeing a dual AChE and BACE-1 inhibitory activity.

## References

[1] Hedden T, Gabrieli JDE. Insights into the ageing mind: a view from cognitive neuroscience. Nat Rev Neurosci 2004;5:87–96. 14735112 10.1038/nrn1323

[2] Hardy J, Selkoe DJ. The amyloid hypothesis of Alzheimer’s disease: progress and problems on the road to therapeutics. Science 2002;297:353–6. 12130773 10.1126/science.1072994

[3] Valko M, Leibfritz D, Moncol J, et al Free radicals and antioxidants in normal physiological functions and human disease. Int J Biochem Cell Biol 2007;39:44–84. 16978905 10.1016/j.biocel.2006.07.001

[4] Cavadas C, Aveleira CA, Souza GF, et al The pathophysiology of defective proteostasis in the hypothalamus – from obesity to ageing. Nat Rev Endocrinol 2016;12:723–33. 27388987 10.1038/nrendo.2016.107

[5] Terry AV, Buccafusco J. The cholinergic hypothesis of age and Alzheimer’s disease-related cognitive deficits: recent challenges and their implications for novel drug development. J Pharmacol Exp Ther 2003;306:821–7. 12805474 10.1124/jpet.102.041616

[6] Eikelenboom P, Veerhuis R, Scheper W, et al The significance of neuroinflammation in understanding Alzheimer’s disease. J Neural Transm 2006;113:1685–95. 17036175 10.1007/s00702-006-0575-6

[7] Selkoe DJ, Schenk D. Alzheimer’s disease: molecular understanding predicts amyloid-based therapeutics. Annu Rev Pharmacol Toxicol 2003;43:545–84. 12415125 10.1146/annurev.pharmtox.43.100901.140248

[8] De Ferrari GV, Canales M, Shin I, et al A structural motif of acetylcholinesterase that promotes amyloid beta-peptide fibril formation. Biochemistry 2001;40:10447–57. 11523986 10.1021/bi0101392

[9] Greig NH, Utsuki T, Ingram DK, et al Selective butyrylcholinesterase inhibition elevates brain acetylcholine, augments learning and lowers Alzheimer beta-amyloid peptide in rodent. Proc Natl Acad Sci USA 2005;102:17213–8. 16275899 10.1073/pnas.0508575102 PMC1288010

[10] Bolognesi ML, Bartolini M, Tarozzi A, et al Multitargeted drugs discovery: balancing anti-amyloid and anticholinesterase capacity in a single chemical entity. Bioorg Med Chem Lett 2011;21:2655–8. 21236667 10.1016/j.bmcl.2010.12.093

[11] Rosini M, Simoni E, Bartolini M, et al Inhibition of acetylcholinesterase, beta-amyloid aggregation, and NMDA receptors in Alzheimer’s disease: a promising direction for the multi-target-directed ligands gold rush. J Med Chem 2008;51:4381–4. 18605718 10.1021/jm800577j

[12] Huang Y, Mucke L. Alzheimer mechanisms and therapeutic strategies. Cell 2012;148:1204–22. 22424230 10.1016/j.cell.2012.02.040 PMC3319071

[13] Zemek F, Drtinova L, Nepovimova E, et al Outcomes of Alzheimer’s disease therapy with acetylcholinesterase inhibitors and memantine. Expert Opin Drug Saf 2014;13:759–74. 24845946 10.1517/14740338.2014.914168

[14] Hansen RA, Gartlehner G, Webb AP, et al Efficacy and safety of donepezil, galantamine, and rivastigmine for the treatment of Alzheimer’s disease: a systematic review and meta-analysis. Clin Interv Aging 2008;3:211–25. 18686744 PMC2546466

[15] Sağlık BN, Ilgın S, Özkay Y. Synthesis of new donepezil analogues and investigation of their effects on cholinesterase enzymes. Eur J Med Chem 2016;124:1026–40. 27783974 10.1016/j.ejmech.2016.10.042

[16] Wang ZM, Cai P, Liu QH, et al Rational modification of donepezil as multifunctional acetylcholinesterase inhibitors for the treatment of Alzheimer’s disease. Eur J Med Chem 2016;123:282–97. 27484514 10.1016/j.ejmech.2016.07.052

[17] Chierrito TPC, Pedersoli-Mantoani S, Roca C, et al From dual binding site acetylcholinesterase inhibitors to allosteric modulators: a new avenue for disease-modifying drugs in Alzheimer’s disease. Eur J Med Chem 2017;139:773–91. 28863358 10.1016/j.ejmech.2017.08.051

[18] Lan JS, Zhang T, Liu Y, et al Design, synthesis and biological activity of novel donepezil derivatives bearing N-benzyl pyridinium moiety as potent and dual binding site acetylcholinesterase inhibitors. Eur J Med Chem 2017;133:184–96. 28388521 10.1016/j.ejmech.2017.02.045

[19] van Greunen DG, Cordier W, Nell M, et al Targeting Alzheimer’s disease by investigating previously unexplored chemical space surrounding the cholinesterase inhibitor donepezil. Eur J Med Chem 2017;127:671–90. 27823887 10.1016/j.ejmech.2016.10.036

[20] Mishra CB, Kumari S, Manral A, et al Design, synthesis, in-silico and biological evaluation of novel donepezil derivatives as multi-target-directed ligands for the treatment of Alzheimer’s disease. Eur J Med Chem 2017;125:736–50. 27721157 10.1016/j.ejmech.2016.09.057

[21] Peauger L, Azzouz R, Gembus V, et al Donepezil-based central acetylcholinesterase inhibitors by means of a “Bio-Oxidizable” prodrug strategy: design, synthesis, and in vitro biological evaluation. J Med Chem 2017;60:5909–26. 28613859 10.1021/acs.jmedchem.7b00702

[22] Azzouz R, Peauger L, Gembus V, et al Novel donepezil-like N-benzylpyridinium salt derivatives as AChE inhibitors and their corresponding dihydropyridine “bio-oxidizable prodrugs: synthesis, biological evaluation and structure–activity relationship”. Eur J Med Chem 2018;145:165–90. 29324339 10.1016/j.ejmech.2017.12.084

[23] Wang J, Wang Z-M, Li X-M, et al Synthesis and evaluation of multi-target-directed ligands for the treatment of Alzheimer’s disease based on the fusion of donepezil and melatonin. Bioorg Med Chem 2016;24:4324–38. 27460699 10.1016/j.bmc.2016.07.025

[24] Li F, Wang ZM, Wu JJ, et al Synthesis and pharmacological evaluation of donepezil-based agents as new cholinesterase/monoamine oxidase inhibitors for the potential application against Alzheimer’s disease. J Enzyme Inhib Med Chem 2016;31:41–53. 27384289 10.1080/14756366.2016.1201814

[25] Wu MY, Esteban G, Brogi S, et al Donepezil-like multifunctional agents: design, synthesis, molecular modeling and biological evaluation. Eur J Med Chem 2016;121:864–79. 26471320 10.1016/j.ejmech.2015.10.001

[26] Xie SS, Lan JS, Wang X, et al Design, synthesis and biological evaluation of novel donepezil-coumarin hybrids as multi-target agents for the treatment of Alzheimer’s disease. Bioorg Med Chem 2016;24:1528–39. 26917219 10.1016/j.bmc.2016.02.023

[27] Prati F, Bergamini C, Fato R, et al Novel 8-hydroxyquinoline derivatives as multitarget compounds for the treatment of Alzheimer’s disease. ChemMedChem 2016;11:1284–95. 26880501 10.1002/cmdc.201600014

[28] Cai P, Fang SQ, Yang XL, et al Rational design and multibiological profiling of novel donepezil-trolox hybrids against Alzheimer’s disease, with cholinergic, antioxidant, neuroprotective, and cognition enhancing properties. ACS Chem Neurosci 2017;8:2496–511. 28806057 10.1021/acschemneuro.7b00257

[29] Dias KS, de Paula CT, Dos Santos T, et al Design, synthesis and evaluation of novel feruloyl-donepezil hybrids as potential multitarget drugs for the treatment of Alzheimer’s disease. Eur J Med Chem 2017;130:440–57. 28282613 10.1016/j.ejmech.2017.02.043

[30] Prati F, Bottegoni G, Bolognesi ML, et al BACE-1 inhibitors: from recent single-target molecules to multitarget compounds for Alzheimer’s disease. J Med Chem 2018;61:619–37. 28749667 10.1021/acs.jmedchem.7b00393

[31] Prati F, Cavalli A, Bolognesi ML. Navigating the chemical space of multitarget-directed ligands: from hybrids to fragments in Alzheimer’s disease. Molecules 2016;21:466. 27070562 10.3390/molecules21040466 PMC6273289

[32] Rampa A, Mancini F, De Simone A, et al From AChE to BACE1 inhibitors: the role of the amine on the indanone scaffold. Bioorg Med Chem Lett 2015;25:2804–8. 26003339 10.1016/j.bmcl.2015.05.002

[33] Piazzi L, Rampa A, Bisi A, et al 3-{4-[(benzylmethylamino)methyl]phenyl}-6,7-dimethoxy-2H-2-chromenone (AP2238) inhibits both acetylcholinesterae and acetylcholinesterase-induced β-amyloid aggregation: a dual function lead for Alzheimer’s disease therapy. J Med Chem 2003;46:2279–82. 12773032 10.1021/jm0340602

[34] Piazzi L, Cavalli A, Belluti F, et al Extensive SAR and computational studies of 3-{4-[(benzylmethylamino)methyl]phenyl}-6,7-dimethoxy-2H-2-chromenone (AP2238) derivatives. J Med Chem 2007;50:4250–4. 17655212 10.1021/jm070100g

[35] Rizzo S, Bartolini M, Ceccarini L, et al Targeting Alzheimer’s disease: novel indanone hybrids bearing a pharmacophoric fragment of AP2238. Bioorg Med Chem 2010;18:1749–60. 20171894 10.1016/j.bmc.2010.01.071

[36] Sugimoto H, Iimura Y, Yamanishi Y, et al Synthesis and structure-activity relationships of acetylcholinesterase inhibitors: 1-benzyl-4-[(5,6-dimethoxy-1-oxoindan-2-yl)methyl]piperidine hydrochloride and related compounds. J Med Chem 1995;38:4821–9. 7490731 10.1021/jm00024a009

[37] Dubey SK, Kharbanda M, Dubey SK, et al A new commercially viable synthetic route for donepezil hydrochloride: anti-Alzheimer’s drug. Chem Pharm Bull 2010;58:1157–60. 20823593 10.1248/cpb.58.1157

[38] Oliverio M, Nardi M, Costanzo P, et al Non-conventional methodologies in the synthesis of 1-indanones. Molecules 2014;19:5599–610. 24786845 10.3390/molecules19055599 PMC6271961

[39] Menezes JCJMDS. Arylidene indanone scaffold: medicinal chemistry and structure–activity relationship view. RSC Adv 2017;7:9357–72.

[40] Costanzo P, Cariati L, Desiderio D, et al Design, synthesis, and evaluation of Donepezil-like compounds as AChE and BACE-1 inhibitors. ACS Med Chem Lett 2016;7:470–5. 27190595 10.1021/acsmedchemlett.5b00483 PMC4867475

[41] Kryger G, Silman I, Sussman JL. Structure of acetylcholinesterase complexed with E2020 (Aricept): implications for the design of new anti-Alzheimer drugs. Structure 1999;7:297–307. 10368299 10.1016/s0969-2126(99)80040-9

[42] Ellman GL, Courtney KD, Andres V, et al A new and rapid colorimetric determination of acetylcholinesterase activity. Biochem Pharmacol 1961;7:88–95. 13726518 10.1016/0006-2952(61)90145-9

[43] Sussman JL, Harel M, Frolow F, et al Purification and crystallization of a dimeric form of acetylcholinesterase from *Torpedo californica* subsequent to solubilization with phosphatidylinositol-specific phospholipase C. J Mol Biol 1988;203:821–3. 2850366 10.1016/0022-2836(88)90213-6

[44] Lausi A, Polentarutti M, Onesti S, et al Status of the crystallography beamlines at Elettra. Eur Phys J plus 2015;130:43.

[45] Leslie AG. The integration of macromolecular diffraction data. Acta Crystallogr. D Biol. Crystallogr 2006;62:48–57. 16369093 10.1107/S0907444905039107

[46] Battye TGG, Kontogiannis L, Johnson O, et al iMOSFLM: a new graphical interface for diffraction-image processing with MOSFLM. Acta Crystallogr D Biol Crystallogr 2011;67:271–81. 21460445 10.1107/S0907444910048675 PMC3069742

[47] Winn MD, Ballard CC, Cowtan KD, et al Overview of the CCP4 suite and current developments. Acta Crystallogr D Biol Crystallogr 2011;67:235–42. 21460441 10.1107/S0907444910045749 PMC3069738

[48] McCoy AJ, Grosse-Kunstleve RW, Adams PD, et al Phaser crystallographic software. J Appl Crystallogr 2007;40:658–74. 19461840 10.1107/S0021889807021206 PMC2483472

[49] Dym O, Song W, Felder C, et al The impact of crystallization conditions on structure-based drug design: a case study on the methylene blue/acetylcholinesterase complex. Prot Sci 2016;25:1096–114. 10.1002/pro.2923 PMC4941771 26990888

[50] Murshudov GN, Skubák P, Lebedev AA, et al REFMAC5 for the refinement of macromolecular crystal structures. Acta Crystallogr D Biol Crystallogr 2011;67:355–67. 21460454 10.1107/S0907444911001314 PMC3069751

[51] Read RJ. Improved Fourier coefficients for maps using phases from partial structures with errors. Acta Crystallogr 1986;A42:140–9.

[52] Emsley P, Lohkamp B, Scott WG, et al Features and development of Coot. Acta Crystallogr. D Biol. Crystallogr 2010;66:486–501. 20383002 10.1107/S0907444910007493 PMC2852313

[53] Morris GM, Huey R, Lindstrom W, et al Autodock4 and AutoDockTools4: automated docking with selective receptor flexiblity. J Comput Chem 2009;16:2785–91. 10.1002/jcc.21256 PMC2760638 19399780

[54] Iserloh U, Wu Y, Cumming JN, et al Potent pyrrolidine- and piperidine-based BACE-1 inhibitors. Bioorg Med Chem Lett 2008;18:414–41. 18023580 10.1016/j.bmcl.2007.10.116

[55] Schüttelkopf AW, van Aalten DMF. PRODRG: a tool for high-throughput crystallography of protein-ligand complexes. Acta Crystallogr D Biol Crystallogr 2004;60:1355–63. 15272157 10.1107/S0907444904011679

[56] Huey R, Garrett MM, Olson AJ, et al A semiempirical free energy force field with charge-based desolvation. J Comput Chem 2007;28:1145–52. 17274016 10.1002/jcc.20634

[57] DeLano WL. PyMOL: An open-source molecular graphics tool. CCP4 Newsletter on Protein Crystallography 2002;40:82–92.

[58] Engh RA, Huber R. Structure quality and target parameters In: Arnold E, Himmel DM, Rossmann MG, eds. International Tables for Crystallography Volume F: Crystallography of biological macromolecules. Chichester: Wiley; 2012:474–84.

[59] Chen VB, Arendall WB, Headd JJ, et al MolProbity: all-atom structure validation for macromolecular crystallography. Acta Crystallogr D Biol Crystallogr 2010;66:12–21. 20057044 10.1107/S0907444909042073 PMC2803126

[60] Kleywegt GJ. Validation of protein models from Cα coordinates alone. J Mol Biol 1997;273:371–6. 9344745 10.1006/jmbi.1997.1309

[61] Harel M, Schalk I, Ehret-Sabatier L, et al Quaternary ligand binding to aromatic residues in the active-site gorge of acetylcholinesterase. Proc Natl Acad Sci USA 1993;90:9031–5. 8415649 10.1073/pnas.90.19.9031 PMC47495

[62] Barate SS, Kumar V, Vishwakarma RA. Determining partition coefficient (Log P), distribution coefficient (Log D) and ionization constant (pKa) in early drug discovery. Comb Chem High Throughput Screen 2016;19:461–9. 27137915 10.2174/1386207319666160502123917

[63] Gallivan JP, Dougherty D. Cation-π interaction in structural biology. Proc Natl Acad Sci USA 1999;96:9459–64. 10449714 10.1073/pnas.96.17.9459 PMC22230

[64] Wallace AC, Laskowski RA, Thornton JM. LIGPLOT: a program to generate schematic diagrams of protein-ligand interactions. Protein Eng 1996;8:127–34. 10.1093/protein/8.2.127 7630882

[65] Raves ML, Harel M, Pang Y-P, et al Structure of acetylcholinesterase complexed with the nootropic alkaloid, (−)-huperzine A. Nat Struct Biol 1997;4:57–63. 8989325 10.1038/nsb0197-57

[66] Koellner G, Kryger G, Millard CB, et al Active-site gorge and buried water molecules in crystal structures of acetylcholinesterase from *Torpedo californica*. J Mol Biol 2000;296:713–35. 10669619 10.1006/jmbi.1999.3468

[67] Saxena A, Fedorko JM, Vinayaka CR, et al Aromatic amino-acid residues at the active and peripheral anionic sites control the binding of E2020 (Aricept) to cholinesterases. Eur J Biochem 2003;270:4447–58. 14622273 10.1046/j.1432-1033.2003.03837.x

